# FakeMusicCaps: A Dataset for Detection and Attribution of Synthetic Music Generated via Text-to-Music Models

**DOI:** 10.3390/jimaging11070242

**Published:** 2025-07-18

**Authors:** Luca Comanducci, Paolo Bestagini, Stefano Tubaro

**Affiliations:** Department of Electronics, Information and Bioengineering (DEIB), Politecnico di Milano, 20133 Milano, Italy; paolo.bestagini@polimi.it (P.B.); stefano.tubaro@polimi.it (S.T.)

**Keywords:** music generation, text-to-music, audio forensics, DeepFake

## Abstract

Text-to-music (TTM) models have recently revolutionized the automatic music generation research field, specifically by being able to generate music that sounds more plausible than all previous state-of-the-art models and by lowering the technical proficiency needed to use them. For these reasons, they have readily started to be adopted for commercial uses and music production practices. This widespread diffusion of TTMs poses several concerns regarding copyright violation and rightful attribution, posing the need of serious consideration of them by the audio forensics community. In this paper, we tackle the problem of detection and attribution of TTM-generated data. We propose a dataset, FakeMusicCaps, that contains several versions of the music-caption pairs dataset MusicCaps regenerated via several state-of-the-art TTM techniques. We evaluate the proposed dataset by performing initial experiments regarding the detection and attribution of TTM-generated audio considering both closed-set and open-set classification.

## 1. Introduction

Deep learning-based music generation [1] has been recently revolutionized by the introduction of text-to-music models. TTM models are usually based on a language model that decodes continuous or discrete tokenized embeddings obtained via some neural audio codec [2,3], such as MusicLM [4], MusicGEN [5], MAGNeT [6], and JASCO [7], or on latent diffusion models operating on some compressed form of audio, such as AudioLDM [8], AudioLDM2 [9], MusicLDM [10], Noise2Music [11], and Mustango [12].

These models are characterized by being able to generate sufficiently realistic music and are simple to use, lowering the technical proficiency needed to successfully interact with them [13]. This combination of factors has made them extremely attractive to the general public and of interest to private industries.

Several commercial TTMs have been proposed, such as Suno [14] (setting the record for the biggest investment ever in an AI music startup, namely $125 million) and Udio [15]. Recently, both companies have been sued by major record companies and have consecutively admitted to copyright infringement, by training their respective models also using unlicensed music. As both the capabilities and commercial interest of these models grow, it is becoming increasingly necessary to begin to develop forensic approaches to be able to detect and analyze music generated via TTMs [16].

The multimedia forensics research field is well mature in image [17,18,19,20,21] and video [22] deepfake detection and model attribution. Concerning audio, forensics approaches have been focusing almost exclusively on speech signals [23,24,25].

In the music domain, most efforts have focused on singing voice detection [26,27,28,29] with the development of specific challenges [30] to foster research in this direction. More specifically, in [26], the authors present SingFake, a dataset for singing voice detection and use it to evaluate four state-of-the-art speech deepfake detectors. In [27], the authors present a dataset of fake chinese songs and analyze the performance of SOTA detectors trained on speech signals and on the proposed dataset. The SingGraph model [28] leverages MERT [31] and wav2vec 2.0 [32] to detect fake singing voices by merging lyrics and audio analysis, while in [29], the authors use singer-level contrastive learning and demonstrate difficulties in detecting cloned-singers.

More recently, other works have tackled the fake music research problem, considering also non-vocal audio parts. An overview of the problem is presented in [33]. In [34], the authors focus on detecting which neural audio codec was used to compress real music tracks and, obtaining surprisingly accurate results, point out several issues that might make the fake music detection problem too easy and rapidly worsen in out-of-domain-scenarios. Moreover, as a detector, they apply a simple convolutional model composed of just six layers, showing that performance is not the only thing to take into account when considering fake music detection. Singing voice is again considered in [35], where it is demonstrated how including background music could enhance the accuracy when classifying fake singing voices. Specifically, the authors apply a hybrid front-end model that extracts features separately from vocals and background music, before feeding the output to a backend network based on Rawnet2 [36] and AASIST [37].

Research in this field is also limited by economic factors, since most models are developed by tech giants that often do not release the code and/or weights. Additionally, available paired text-music datasets are scarce. Notable example datasets include MusicCaps [4], containing 5500 musiclips extracted from AudioSet [38] and annotated by human musicians, Song Describer [39], containing 1100 human-made captions of 706 music recordings, MusicBench [12], which contains music obtained by augmenting and modifying the MusicCaps dataset, obtaining 52,000 samples, and the recently proposed JamendoMaxCaps [40], where 362,000 captions from the Jamendo dataset are described using an audio captioning system [41].

In this paper, we propose the FakeMusicCaps dataset, with the objective of encouraging research in the detection of music deepfakes. To build FakeMusicCaps, we replicate the MusicCaps dataset by using its captions as input to five state-of-the-art TTM models, namely MusicGen [5], MusicLDM [10], AudioLDM2 [9], Stable Audio Open [42], and Mustango [12]. The nature of FakeMusicCaps makes it easy to incorporate future TTMs by simply generating music examples using the same procedure. We perform a simple benchmark study, on FakeMusicCaps, by studying if it is possible to perform detection and attribution, i.e., classifying the input music as either real or belonging to one of the chosen TTM models, using state-of-the-art models. We analyze how the models perform both in closed set and open set scenarios, where the latter also includes data belonging to generators not seen during training, specifically, belonging to the SunoCaps dataset [43].

At the same time of this work, a similar dataset, named SONICS, has been proposed [44], which however considers only the commercially-available models Suno and Udio and performs only real/fake music detection. More specifically, in [44] the authors explore the classification of real and fake music by proposing the Spectro-Temporal Tokens Transformer (SpecTTTra) architecture to perform fake music detection.

We instead focus on open-source TTMs and consider commercial ones only in the open set classification. The reasoning behind this is that open-source techniques are possibly available to a wider part of the research community, which could use them to integrate FakeMusicCaps as they see fit. Moreover, open-source models allow researchers to have complete knowledge of the entire pipeline used to generate the audio tracks, enabling them to make stronger assumptions on the behaviors observed when classifying the data. This is particularly useful when dealing with open-set scenarios, since without knowing the audio generation process it is impossible to deem audio signals as belonging to different generators.

Since its release, the FakeMusicCaps dataset has already been used to foster the research in fake music detection. In fact, in [45], the authors performed a study aimed at understanding the behavior of classifiers operating on the FakeMusicCaps dataset, by applying eXplainable Artificial Intelligence (XAI) techniques. Inspired by FakeMusicCaps and SONICS, the authors of [46] propose the M6 dataset, which aggregates various types of audio content from existing datasets for what concerns real music and generates fake music using simple custom prompts.

Our contributions can then be summarized as follows:

- In this paper, we release FakeMusicCaps, the first dataset specifically designed for both detection and attribution of fake music. The dataset is created using only open-source text-to-music models, making the generation process fully transparent.
- Through the use of simple network architectures, we analyze the detection and (for the first time) attribution of fake music generated via TTM models. We consider both closed-set and open-set classification scenarios, taking into account models generated via Suno in the latter.

The remainder of the paper is organized as follows. In Section 2, we introduce the attribution problem for TTM models. In Section 3, we describe how the FakeMusicCaps dataset was created. Section 4 presents the experimental setup used to conduct the experiments, while in Section 5, we present the results to analyze the complexity of TTM attribution and the effectiveness of the proposed dataset. Finally, in Section 7, we draw some conclusions. The code used to generate FakeMusicCaps and perform the experiments (https://github.com/polimi-ispl/FakeMusicCaps, accessed on 13 July 2025) as well as the complete dataset (https://zenodo.org/records/15063698, accessed on 13 July 2025) are publicly available.

## 2. Problem Formulation

Given some kind of text representation τ and a composite model T(·), the TTM techniques model the function x=T(τ), where $x\in R^{1\times N}$ is an audio waveform containing music that corresponds to the textual description provided in τ.

**Figure 1:**
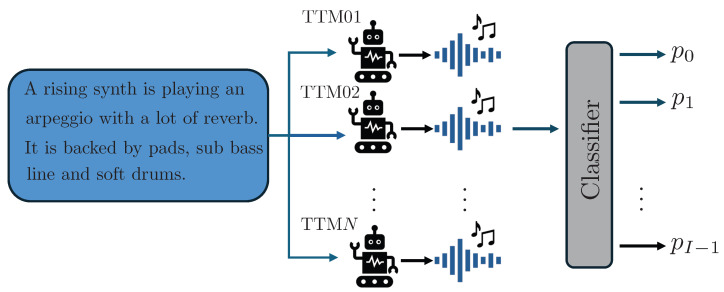
Schematic representation of the text-to-music attribution problem.

The text-to-music attribution problem, schematically shown in Figure 1, can be formally defined as follows. Given the discrete-time music signal x and a set of *I* TTM models $\left\{T_{0},\ldots ,T_{I-1}\right\}$, the objective is to determine which generator $T_{i}$ has been used to generate x. This is done by training a classifier that takes as input x and outputs the probabilities $p_{i},i=0,\ldots ,I-1$ of x generated using each of the known TTM models.

The attribution problem is often considered both in closed- and open-set scenarios. In the former, all generators are seen both during training and testing, while in the latter, some TTMs are unknown during training and seen only at testing time, posing the need to develop specific classification strategies.

## 3. FakeMusicCaps Dataset

**Figure 2:**
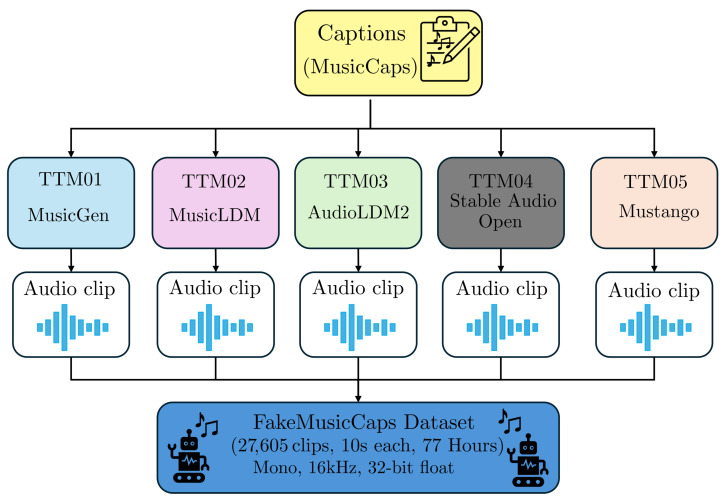
Flowchart summarizing the creation of the FakeMusicCaps dataset.

In this section, we describe how the FakeMusicCaps dataset was created, first by presenting the chosen TTM models and then describing the generation procedure. We schematically represent the creation of the FakeMusicCaps dataset in Figure 2.

To create FakeMusicCaps, we take the MusicCaps evaluation dataset [4] and use its captions as input to five state-of-the-art TTM models, which we will describe in the following. The original audio files contained in the MusicCaps dataset are actual music clips extracted from the AudioSet dataset [47] and described using a free text caption by musicians. The characteristics of the audio content in terms of musical instruments, timbre, and variety, the FakeMusicCaps dataset depends on the dataset on which each TTM model was trained, and on the peculiarity of each of these models.

### 3.1. Considered Architectures

In this section, we present an overview of the architectures (TTM01-TTM05) used to create the FakeMusicCaps dataset.

- **TTM01-MusicGen** [5] is an autoregressive language model, based on a single-stage transformer that decodes discrete audio tokens obtained via Encodec [3]. It was trained over an undisclosed dataset of over 20,000 h of music. We use the *medium* checkpoint consisting of 1.5 *B* parameters.
- **TTM02-MusicLDM** [10] is a latent diffusion model operating on compressed audio representations extracted via HiFi-GAN [48]. It adapts AudioLDM to the musical domain, by introducing beat-synchronous audio mixup and beat-synchronous latent mixup strategies, to augment the quantity of data used for training. The text conditioning is provided via CLAP [49], which the authors fine-tune on music for a total of 20,000 h. The MusicLDM model is then trained on the Audiostock dataset [49], containing 455.6 h of music.
- **TTM03-AudioLDM2** [9] is a latent diffusion model where the audio is compressed via a Variational AutoEncoder (VAE) and HiFiGAN, similarly to the AudioLDM pipeline. However, the major difference of AudioLDM2 with respect to the previous version, is that the diffusion model is conditioned through AudioMAE [50] that enables the adoption of a “Language of Audio”, to generate a wide variety of types of audio. We use the *audioldm2-music* checkpoint to build FakeMusicCaps, specifically trained for text-to-music generation.
- **TTM04-Stable Audio Open** [42] is a latent-diffusion architecture generating stereo data at 44.1kHz based on a variant of Stable Audio [51] that uses T5 [52] as a text encoder. The model is trained only on Creative Commons-licensed audio data for a total of 7.3 K hours of audio.
- **TTM05-Mustango** [12] is a diffusion-based TTM model that through a Music-domain-knowledge-informed UNet (MuNet) injects music concepts such as chord, beats, key, or tempo in the generated music, during the reverse diffusion process. Through data augmentation, the authors generate the MusicBench dataset, composed of 53.168 tracks, to train the model. The model generates at 16kHz

### 3.2. Generation Strategy

In this section, we describe the strategy used to generate the FakeMusicCaps dataset.

We derive the inspiration for the generation procedure from the MusicCaps [4] dataset, consisting of 5500 music clips, each 10 s long, extracted from AudioSet [38]. Each track is supplied with an annotation by a professional musician. MusicCaps has rapidly become the benchmark dataset for the evaluation of TTM models.

To create FakeMusicCaps, we use the captions from MusicCaps, and for each one of them, we generate a corresponding 10 s audio track using models (TTM01-TTM05) for a total of 27,605 music tracks corresponding to almost 77 h.

Since the objective of the dataset is to provide an initial dataset for the analysis of the detection and attribution of music generated via TTM models, we adopt an audio pipeline that ensures that all audios are represented using the same format. Specifically, each track is first converted to mono and downsampled to the sampling frequency $F_{s}=16\;\mathrm{kHz}$. Finally, we save each track using the 32-bit float wav format.

## 4. Experimental Analysis

In this section, we describe the experiments performed with the aim of showing a first validation of the FakeMusicCaps dataset, considering both closed set and open set scenarios.

### 4.1. Dataset

We used the FakeMusicCaps dataset during the training and test procedures. We made sure that the training and test datasets were disjoint. Specifically, we built the test set by selecting 320 tracks from FakeMusicCaps, as a selection criterion, for each TTM model, we chose those having the same captions as the SunoCaps [43] dataset. This choice was operated in order to be able to coherently use the Suno-generated music excerpts from SunoCaps to perform the open-set scenario experiments.

### 4.2. Baselines

We use three classification models as simple benchmarks of the FakeMusicCaps for deepfake music detection and attribution.

We first consider a very simple network that operates on raw audio, namely M5 [53]. This network consists of only 0.5 M parameters and leverages the adoption of several consecutive layers. We use this simple network as an initial experiment, in order to understand the level of hardness of the music deepfake detection and attribution problem.

Then, we selected a more complicated method operating on raw audio, namely RawNet2 [36]. This model, is an end-to-end neural network that has been used as a baseline for several antispoofing challenges such as ASVspoof 2021 and consists of Sinc Filters, followed by residual blocks and a Gated Recurrent Unit (GRU).

We also consider a model operating on log-spectrograms, namely ResNet18+Spec [54]. This model is a modified version of ResNet18, consisting of 18-layer deep convolutional layer with residual connections. The modifications make it suitable to work with 1-channel log-spectrograms.

All methods were modified by adding a fully connected layer with a number of neurons corresponding to the number of considered classes at the end of each network. This modification is necessary in order to be able to discriminate correctly between the considered TTM models.

### 4.3. Training

All models were trained to discriminate between 6 different classes, comprising the 5 known TTM models and the real music signals belonging to MusicCaps.

We trained all models using cross-entropy as a loss function and the Adam optimizer with a learning rate of 1 × 10^−4^.

All networks were trained for a maximum of 100 epochs, ending the training earlier if the loss did not improve for more than 10 consecutive epochs. We used a batch size of 32 for M5 and 16 for RawNet2 and ResNet18 + Spec. In the case of ResNet18 + Spec, we computed the STFT with 512 frequency points, using a Hann window of length 512 samples with a hop size of 128 samples.

### 4.4. Classification Techniques

In the *closed set* classification problem, given a raw audio waveform corresponding to music, we want to identify the generation method from the set of *known* (i.e., a set of models included in the training dataset) TTM models.

Differently, in the case of *open set* classification, we want also to determine if some audio tracks belong to a TTM model that it is *unknown*, i.e., not included in the training dataset.

If we consider $p_{i}$ as the output of the softmax layer of the models, then in the closed set case, class attribution can simply be performed by computing $\arg\max_{i}p_{i}$. For open set classification, instead, we follow two different approaches. In the open set *(threshold)* technique [55], we compute the two highest values of $p_{i}$, defined as $p_{1}$ and $p_{2}$, and then classify the input example as unknown if the ratio $p_{1}/p_{2}$ between these values is higher than a threshold. The rationale is that, if the TTM method used to generate the audio track was known from the training set, only one $p_{i}$ value should be high. More formally, the predicted TTM model $\hat{T}$ can be obtained as

$$\hat{T}=\left\{\begin{array}{cc} \arg\max_{i}p_{i} & \mathrm{if}\frac{p_{1}}{p_{2}}>\gamma ,\\ I+1, & \mathrm{otherwise}, \end{array}\right.$$

where γ is a threshold that should be determined empirically, following [24], we choose γ=2.

In the open set *SVM* technique, instead, we train a one-class Support Vector Machine (SVM), using radial basis functions kernel, over the $p_{i}$ values computed from the training data. The output of the classification is binary: either the class is known or not.

## 5. Results

In this section, we present preliminary results aimed at demonstrating the suitability of FakeMusicCaps as an initial dataset for text-to-music model detection and attribution. Specifically, we test the performance of state-of-the art models for fake music detection and attribution, both in the closed- and open-set scenarios. Then we perform an additional experiment aimed at understanding the impact of the length of the considered audio tracks.

More specifically, in Section 5.1, we consider the simpler scenario where the TTM seen during training and test phases are the same. In Section 5.2, instead, we consider the more challenging and realistic open-set scenario where the detection models are trained on TTM01, TTM02, TTM03, TTM03, TTM04, and TTM05 and are tested on the SunoCaps [43] dataset, whose audio files are generated using the commercial TTM model Suno, which is not used to generate audio files used during training.

### 5.1. Closed-Set Performances

Despite closed-set classification on a single dataset is often considered a trivial task in forensic applications, it is worth investigating the performance of the tested methods in this scenario.

**Figure 3:**
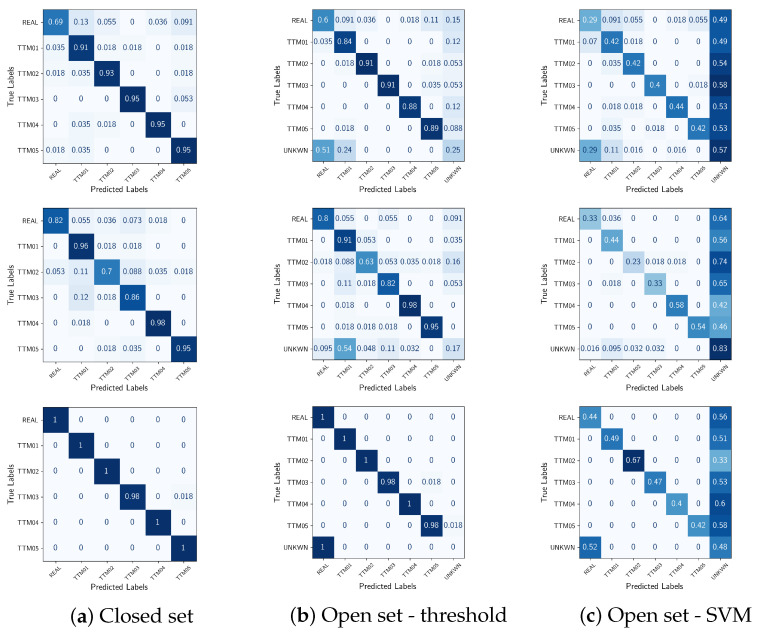
Confusion matrices of M5 (**top**), RawNet2 (**middle**) and ResNet+Spec (**bottom**) in the three classification scenarios.

Table 1 reports closed-set classification results in terms of balanced accuracy ${\mathrm{ACC}}_{B}$ [56], Precision, Recall, and F1 Score. Additionally, the left column of Figure 3 shows the confusion matrix corresponding to M5, RawNet2 and ResNet18+Spec, respectively.

In all metrics, ResNet18 + Spec provides the best performance, while RawNet2 obtains slightly worse results than M5. From the inspection of the confusion matrices, we can see that ResNet18 slightly confounds TTM03 (AudioLDM2) with TTM05 (Mustango), it is interesting to notice that both are diffusion-based models. M5 has a slightly lower performance in detecting the ground-truth data, while RawNet2 struggles more to detect model TTM02 (MusicLDM).

### 5.2. Open Set Performances

We show in Table 2 the performance obtained when performing open set classification using the threshold approach. The corresponding confusion matrices are shown in the second column of Figure 3.

Results corresponding to the open set classification using the SVM approach are shown in Table 3. The corresponding confusion matrices are shown in the last column of Figure 3.

As expected, the open-set scenario is much more challenging than the closed-set one for all the classification models considered. If we look at the results reported in Table 2 and Table 3, we can see that again ResNet18 + Spec achieves the best performance in both cases and that the results obtained via the SVM technique are much worse than the ones obtained via the thresholding approach. However, the analysis of the results becomes different if we look at the confusion matrices. When considering the thresholding method (corresponding to the middle column), we can see that ResNet18 + Spec obtains the best performance when classifying the known methods, but misclassifies all audio excerpts belonging to the class not seen during training and named *UNKNWN* in the image. Interestingly enough, these are confounded with the real examples, which is somewhat expected, given that the commercial model Suno is probably the most realistic of the considered TTM models. M5 and RawNet2 obtain somehow a similar performance, with the former confounding UNKNWN examples with real and MusicGen-generated ones, while the latter mostly confounding them with MusicGen.

In the case of the SVM open-set approach, all models behave differently. Approximately half the time, the classification models mistake the known TTM techniques for the unknown one. Interestingly, RawNet2 obtains the highest accuracy of 0.82 for what concerns the unknown class, and even in this case, ResNet18 mistakes it for the real one.

While more complicated techniques for open-set classification could be used [57,58], the results included here are only intended to provide an initial benchmark of tackling the fake music detection problem on the FakeMusicCaps dataset. More complicated approaches will be considered in future works.

### 5.3. Impact of Window Size

We also performed a small experiment to verify how much the impact of the temporal window length used as input to the models changes their performance. This is important, especially for the design of further datasets, i.e., do we need to create longer musical excerpts or not?

**Figure 4:**
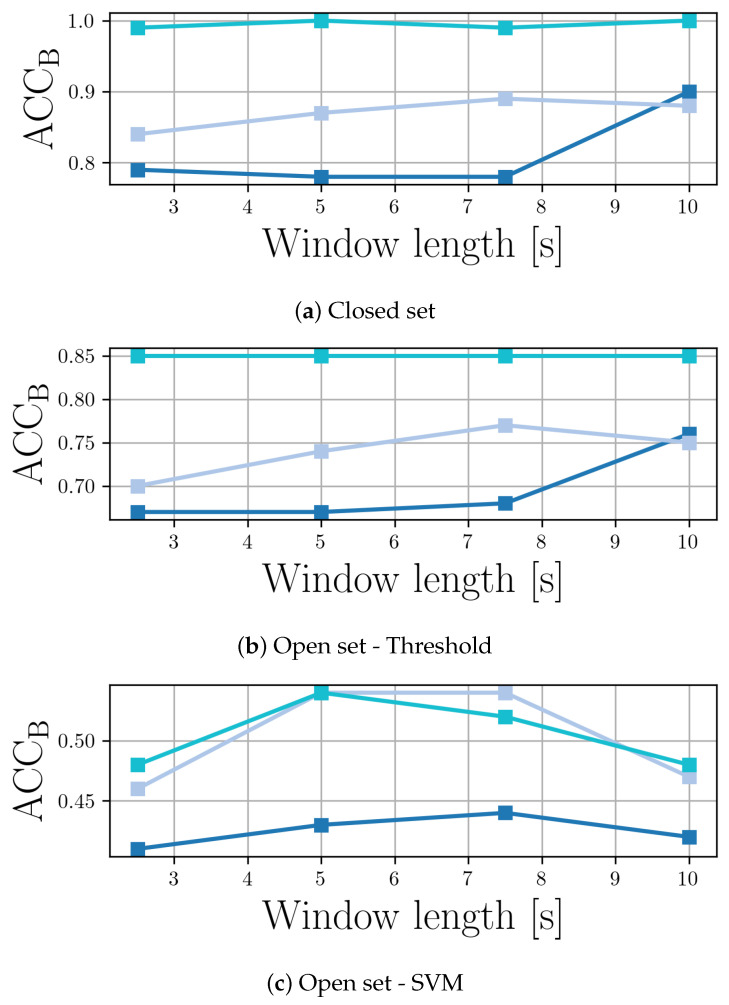
Balanced accuracy varying according to the considered window size using M5 (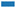), RawNet2 (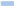), and ResNet + Spec (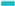).

We consider four window lengths, namely 10s, 7.5s, 5 s, and 2.5s and report the results in terms of balanced accuracy in Figure 4. As we can see, the variations in accuracy are not extreme in all classification scenarios. M5 seems to have an increase in accuracy passing from 7.5 to 10 s window length for both closed set and open set (threshold) methods. ResNet18+Spec does not have major improvements, while a slight increase in accuracy is seen for RawNet2. Results in the case of the Open set (SVM) show a less clear trend, but the impact of the window size does not seem to be relevant even in this case.

## 6. Discussion

The objective of the results provided in this paper is to present a first approach to the TTM model detection and attribution and do not claim at all to be definitive. Instead, we hope to further motivate research in this direction. New TTM models are proposed almost monthly if not daily, with a continuous increase in quality, especially concerning commercial models.

For these reasons, while from the results indicated in this paper the problem may seem to be relatively easy, especially in the closed-set scenario, things are not going to stay that way for long, and the research community needs to prepare in advance to solve problems related to the detection of fake music. We can already identify some developments not analyzed in this paper that could be considered in future works related to TTM attribution.

For example, *Do the textual descriptions have an effect on the classification performance?* If text and music are effectively mutually dependent, in the scenario of TTM models, we could be able to leverage that.

Additionally, *can we leverage music theory and musicology to detect music deepfakes?* The analysis of musical theory could be of interest in a context where generated music is inserted in otherwise “real” music, a problem denoted as *splicing.*

Moreover, the widespread diffusion of fake music also poses some practical problems. Fake music detectors should be easily deployable on on-line streaming services and in any case where music is streamed live. In order to be able to do this, it is important to create models that are both lightweight enough to be usable in such scenarios and scalable in terms of inference to a high quantity of data.

## 7. Conclusions

In this paper, we tackled the problem of detecting and attributing music generated via text-to-music models. Specifically, we introduced the FakeMusicCaps dataset, created by replicating the MusicCaps dataset via five state-of-the-art TTM models. By applying simple audio forensics techniques, we demonstrate that the dataset could be used as an initial benchmark to tackle TTM detection and attribution. Future developments will also include extending the dataset to contain captions from datasets other than MusicCaps. Our results are not to be considered definitive, instead, our objective is to further motivate the research in forensics techniques for the analysis of generated music. In fact, while the problem of fake music detection and attribution is now relatively simple, it is guaranteed to grow more extremely complicated day by day. 

## Figures and Tables

**Table 1:** Closed-set classification performances.

Model	${\mathrm{ACC}}_{B}↓$	Precision	Recall	F1 Score
M5	0.90	0.90	0.90	0.90
RawNet2	0.88	0.89	0.88	0.88
ResNet18 + Spec	1.00	1.00	1.00	1.00

**Table 2:** Open set (Threshold) classification performances.

Model	${\mathrm{ACC}}_{B}↓$	Precision	Recall	F1 Score
M5	0.76	0.76	0.76	0.75
RawNet2	0.75	0.75	0.75	0.74
ResNet18 + Spec	**0.85 **	**0.78**	**0.85**	**0.8**

**Table 3:** Open set (SVM) classification performances.

Model	${\mathrm{ACC}}_{B}↓$	Precision	Recall	F1 Score
M5	0.42	0.67	0.42	0.48
RawNet2	0.47	0.80	0.47	0.52
ResNet18 + Spec	**0.48**	**0.80**	**0.48**	**0.56**

## Data Availability

The original dataset presented in the study is openly available at https://zenodo.org/records/15063698, accessed on 13 July 2025, while the code used to perform the experiments is available at https://github.com/polimi-ispl/FakeMusicCaps, accessed on 13 July 2025.
